# Genotype-Phenotype Comparison in POGZ-Related Neurodevelopmental Disorders by Using Clinical Scoring

**DOI:** 10.3390/genes13010154

**Published:** 2022-01-15

**Authors:** Dóra Nagy, Sarah Verheyen, Kristen M. Wigby, Artem Borovikov, Artem Sharkov, Valerie Slegesky, Austin Larson, Christina Fagerberg, Charlotte Brasch-Andersen, Maria Kibæk, Ingrid Bader, Rebecca Hernan, Frances A. High, Wendy K. Chung, Jolanda H. Schieving, Jana Behunova, Mateja Smogavec, Franco Laccone, Martina Witsch-Baumgartner, Joachim Zobel, Hans-Christoph Duba, Denisa Weis

**Affiliations:** 1 Institute of Medical Genetics, Kepler University Hospital Med Campus IV, Johannes Kepler University Linz, A-4020 Linz, Austria; hans-christoph.duba@kepleruniklinikum.at (H.-C.D.); denisa.weis@kepleruniklinikum.at (D.W.); 2 Institute of Human Genetics, Diagnostic and Research Center for Molecular BioMedicine, Medical University of Graz, 8010 Graz, Austria; sarah.verheyen@medunigraz.at; 3 Department of Pediatrics, University of California, San Diego, CA 92161, USA; kwigby@health.ucsd.edu; 4 Rady Children’s Hospital-San Diego, Rady Children’s Institute for Genomic Medicine, San Diego, CA 92123, USA; 5 Research Centre for Medical Genetics, 115478 Moscow, Russia; borovikov@med-gen.ru; 6 Veltischev Research and Clinical Institute for Pediatrics, Pirogov Russian National Research Medical University, 125412 Moscow, Russia; a.a.sharkov@yandex.ru; 7 Department of Pediatrics, Section of Clinical Genetics and Metabolism, Children’s Hospital Colorado, University of Colorado School of Medicine, Aurora, CO 80045, USA; valerie.slegesky@childrenscolorado.org (V.S.); austin.larson@childrenscolorado.org (A.L.); 8 Department of Clinical Genetics, Odense University Hospital, 5000 Odense, Denmark; christina.fagerberg@rsyd.dk (C.F.); Charlotte.B.Andersen@rsyd.dk (C.B.-A.); 9 Institute of Clinical Research, Faculty of Health Sciences, University of Southern Denmark, 5000 Odense, Denmark; 10 H C Andersen Children’s Hospital, Odense University Hospital, 5000 Odense, Denmark; maria.kibaek@rsyd.dk; 11 Institute of Human Genetics, University Hospital, Salzburger Landeskliniken and Paracelsus Medical University Salzburg, A-5020 Salzburg, Austria; i.bader@salk.at; 12 Department of Pediatrics, Columbia University, Irving Medical Center, New York, NY 10032, USA; rh2813@cumc.columbia.edu; 13 Division of Genetics, Massachusetts General Hospital, Boston, MA 02114, USA; fhigh@partners.org; 14 Department of Surgery, Boston Children’s Hospital, Boston, MA 02115, USA; 15 Department of Pediatrics and Medicine, Columbia University, New York, NY 10032, USA; wkc15@cumc.columbia.edu; 16 Department of Pediatric Neurology, Radboud University Hospital Nijmegen, 6525 Nijmegen, The Netherlands; jolanda.schieving@radboudumc.nl; 17 Institute of Medical Genetics, Medical University of Vienna, 1090 Vienna, Austria; jana.behunova@meduniwien.ac.at (J.B.); mateja.smogavec@meduniwien.ac.at (M.S.); franco.laccone@meduniwien.ac.at (F.L.); 18 Division of Human Genetics, Medical University Innsbruck, 6020 Innsbruck, Austria; witsch-baumgartner@i-med.ac.at; 19 Department of Pediatrics, Division of General Pediatrics, Medical University of Graz, 8036 Graz, Austria; joachim.zobel@medunigraz.at

**Keywords:** *POGZ* gene, neurodevelopmental disorder, White-Sutton syndrome, genotype-phenotype association, clinical scoring, deep facial gestalt analysis, nonsense-mediated RNA decay

## Abstract

*POGZ*-related disorders (also known as White-Sutton syndrome) encompass a wide range of neurocognitive abnormalities and other accompanying anomalies. Disease severity varies widely among *POGZ* patients and studies investigating genotype-phenotype association are scarce. Therefore, our aim was to collect data on previously unreported *POGZ* patients and perform a large-scale phenotype-genotype comparison from published data. Overall, 117 *POGZ* patients’ genotype and phenotype data were included in the analysis, including 12 novel patients. A severity scoring system was developed for the comparison. Mild and severe phenotypes were compared with the types and location of the variants and the predicted presence or absence of nonsense-mediated RNA decay (NMD). Missense variants were more often associated with mild phenotypes (*p* = 0.0421) and truncating variants predicted to escape NMD presented with more severe phenotypes (*p* < 0.0001). Within this group, variants in the prolin-rich region of the POGZ protein were associated with the most severe phenotypes (*p* = 0.0004). Our study suggests that gain-of-function or dominant negative effect through escaping NMD and the location of the variants in the prolin-rich domain of the protein may play an important role in the severity of manifestations of *POGZ*–associated neurodevelopmental disorders.

## 1. Introduction

The POGZ protein (pogo transposable element-derived protein with zinc finger domain) is a heterochromatin protein 1α (HP1α)-binding protein, which destabilizes the interaction between HP1α and chromatin and dissociates Aurora B kinase from the chromosome arm during meiosis [1]. POGZ also interacts with SP1 transcription factor and chromodomain helicase DNA-binding protein 4, which suggests that POGZ functions as a chromatin regulator, and thus is important for the normal mitotic progression, especially during cortical development [2]. Analyses of *pogz* knockout mice revealed that the POGZ protein promotes chromatin accessibility and expression of clustered synapse genes, and co-occupies loci with *ADNP*, a gene associated with autism [3].

POGZ dysfunction leads to a wide spectrum of neurodevelopmental disorders, also referred to as White-Sutton syndrome [4,5]. The clinical spectrum includes developmental delay, autism-spectrum disorders and other neuropsychiatric problems with or without structural brain malformations, mild-to-severe intellectual disability, seizures, visual impairment, hearing loss, gastrointestinal and urinary tract anomalies. The expressivity of *POGZ*-related disorder is variable. Multiple types of variants, including missense, nonsense and frameshift variants and deletions have been identified in *POGZ* patients. Missense variants appear to be associated with behavioral anomalies rather than intellectual disability [6], while nonsense and frameshift variants which escape nonsense-mediated RNA decay (NMD) are more likely to cause intellectual disability and accompanying malformations of the gastrointestinal or urinary tract [5]. However, a clear genotype-phenotype correlation has not yet been identified.

Our aim was to further delineate the clinical and genotype spectrum of *POGZ*-associated disorders with novel cases, and to perform a genotype-phenotype comparison based on clinical scoring established from published data.

## 2. Subjects and Methods

Overall, 13 patients (12 novel) with *POGZ*-related neurodevelopmental disorders were recruited in this study with the help of the GeneMatcher platform [7]. Patients and/or legal representatives gave their informed consent to the study. Enrolled patients presented with different severity of the disease and 12 different genotypes. For variant classification Varsome, based on ACMG Guidelines, ClinVar and LOVD Databases were used [8,9,10,11]. The GnomAD Database was applied to assess variant frequency in the general population [12]. In order to predict whether the nonsense and frameshift variants escape nonsense-mediated RNA-decay, we used the NMDEscPredictor computational tool [13].

**Figure 1:**
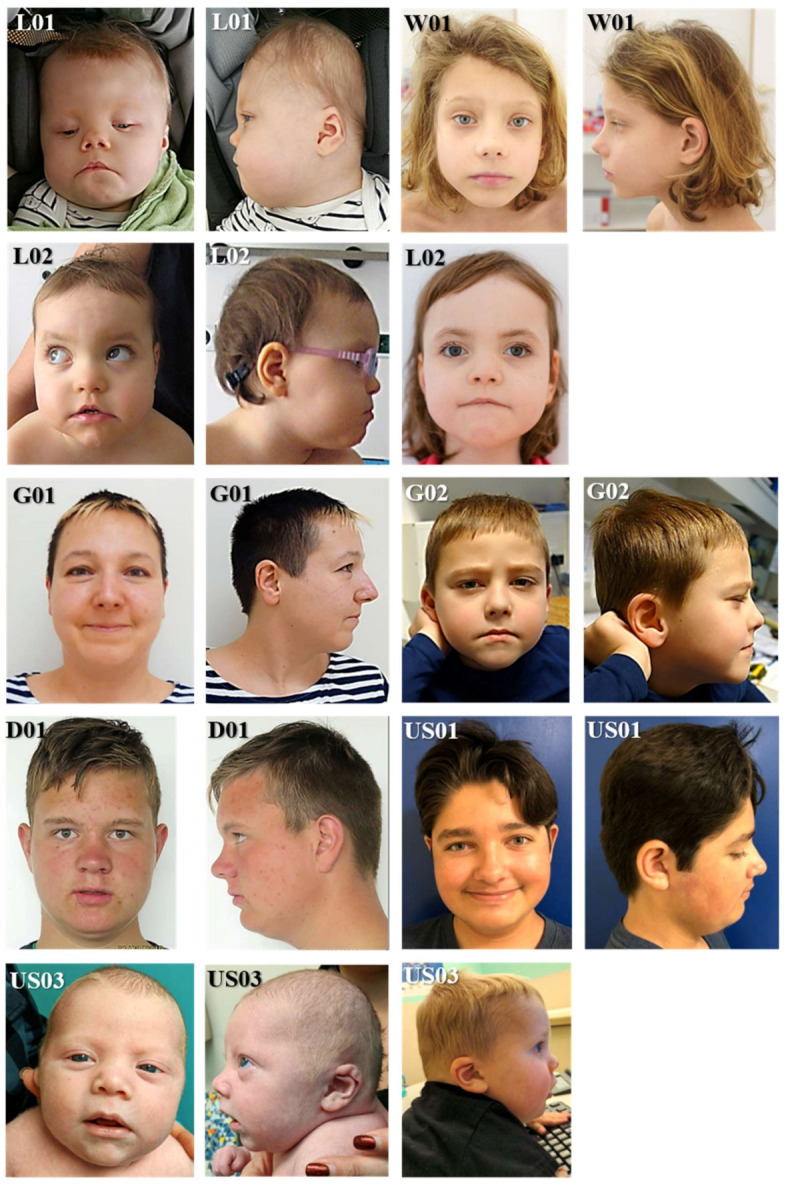
Pictures of *POGZ* patients enrolled in the present study. Patient L01 at the age of 11 months, W01: at the age of 9 years, L02: at the age of 1 year and 4 years, G01: at the age of 35 years, G02: at the age of 5 years, D01: at the age of 17 years, US01: at the age of 14 years, US03: in infancy and at the age of 2.5 years.

The detailed features and abnormalities of our patients are presented in Figure 1 and Supplementary Table S1. For phenotype-genotype comparison, a detailed questionnaire about all the described symptoms and anomalies related to the *POGZ* gene was created and filled out by the patients’ pediatrician/geneticist/genetic counsellor.

### 2.1. Clinical Evaluation and Severity Scoring

We collected all case reports, reviews and cohort studies about *POGZ* patients published until November 2021. The articles were assessed for genotype and phenotype data about *POGZ* patients. The clinical descriptions varied widely in phenotypic details and elaboration. Fourteen articles contained detailed clinical data of about 36 patients [4,14,15,16,17,18,19,20,21,22,23,24,25,26] and six articles contained less detailed phenotypes about 68 patients [5,6,27,28,29,30] (Supplementary Table S1). Overall, 117 patients were included in the analysis (62 males, 53 females, gender not reported in two patients; age range at the genetic diagnosis: prenatally–36 years, mean age and median of the age at the genetic diagnosis: 8 years and 6 years, respectively).

The symptoms were evaluated according to the observed dysmorphic features and abnormalities of the organ systems, such as nervous system, musculature, urinary or gastrointestinal tract. An individual scoring system was set up for each organ system, based on the number of symptoms (Table 1). The highest clinical scores belonged to the abnormalities of the nervous system, cognitive and motor development, followed by the skeletal system (including micro-, brachycephaly), the digestive system, ocular abnormalities and the genitourinary tract (Table 1). Each patient received a cumulative clinical score from the systemic scores. In order to be able to uniformly assess and compare the phenotypes, the cumulative clinical scores were converted into uniform severity scores. Severity score 1 was interpreted as the mildest phenotype, with score 2 as moderate, score 3 as moderate-severe and score 4 as the most severe (Table 2). The patients were grouped into three cohorts, based on the elaboration of the available clinical data. Cohort 1 contained our patients with the most available clinical data, except for patient US02, who was regrouped into cohort 3, due to young age and lack of clinical data. Cohort 2 comprised patients from the literature with detailed phenotypic data, covering all or almost all of our evaluation criteria and cohort 3 patients with less detailed phenotypes, covering fewer evaluation criteria. In each cohort the cumulative clinical scores were grouped into four categories to obtain the unified severity score from 1 to 4 (Table 2, Supplementary Table S1).

Each *POGZ*-related symptom was sorted by frequency in cohort 1 and 2. Cohort 3 was excluded from this analysis due to the lack of information about several *POGZ*-related symptoms (Supplementary Table S1).

### 2.2. Variant Evaluation

The reported variants were grouped according to variant type, whether they are predicted to undergo or escape nonsense-mediated mRNA decay (NMD), and according to their location in the gene and domains. POGZ protein contains 14 domains: zinc finger domains 1–8 (ZF 1–8) and HP1α-binding zinc-finger-like domain (HPZ: zinc finger domain 9), followed by a prolin-rich, centromere protein (CENP)-B-DNA-binding domain (CENPB), transposase encoded DDE, coiled coil domain and an integrase domain-binding motif [1,5].

### 2.3. Facial Gestalt Analysis

For the facial dysmorphology comparison we used DeepGestalt technology [31] via Face2Gene application (FDNA, Inc., Boston, MA, USA). Only patients with a photo of sufficient quality were enrolled in the analysis. A total of 48 photos from patients with all severity scores were included. For the facial analysis each cohort had to comprise at least 10 photos. The cohort with severity score 1 contained only four photos. Therefore, *POGZ* patients were regrouped into two cohorts: mild and severe. The cohort defined as having a mild phenotype consisted of 21 *POGZ* patients with severity score 1 and 2 and the cohort with severe phenotypes contained 27 patients with severity score 3 and 4. The two cohorts were compared to each other and also to the cohort of 79 healthy individuals in a binary comparison and as composite photos. Healthy controls showed no obvious syndrome-related dysmorphism, and were never suspected to have any genetic syndrome. In the binary comparison, a mean area under the curve (AUC) value was generated, which represented the degree of discrimination between the cohorts. Mean AUC ranged between 0 and 1 (0: incorrectly classified cohorts, 0.5: random classification and 1: perfect separation between the cohorts). The *p* value describes the accuracy of the binary comparison, and *p* < 0.05 was considered to be statistically significant, indicating that the Face2Gene software is able to distinguish between the two cohorts.

### 2.4. Statistical Analysis

The GraphPad Prism version 6.01 for Windows software (GraphPad Software, San Diego, CA, USA) was used. The Fisher′s exact test and Chi-squared test with Yates′ correction was performed to compare the variant types and the characteristics between patient cohort with mild phenotype (severity score 1 and 2) and severe phenotypes (severity score 3 and 4). *p* < 0.05 was considered to be statistically significant. Severity scores were expressed as means ± standard deviation (means ± SD) in the different gene regions and domains.

## 3. Results

### 3.1. Variant Types and Their Distribution

Eight variants were novel in our patient cohort, including the whole *POGZ* gene deletion (Table 3). All variants where inheritance could be established were de novo of origin, except one case, where a nonsense variant was maternally inherited (G01 and G02). Both mother and child were presented with mildly delayed development and only a few dysmorphic features, thus representing a mild phenotype.

The total 117 patients carried 72 different variants. Nineteen variants recurred two or more times in overall 45 patients. Multiple variant types were identified including nonsense (*n* = 48, 41%), frameshift (*n* = 47, 40%), missense (*n* = 10, 8.5%), splice site variants (*n* = 8, 7%), larger deletions encompassing several exons or the whole gene (*n* = 3, 2.5%) and small in frame deletion (*n* = 1, 1%). All patients carrying the same recurrent variant presented with the same disease severity, except for three variants. The discrepant cases belonged to cohort 3, with a less detailed phenotype (Supplementary Table S1).

Segregation analysis was performed in 82 cases. In 74 cases (90%) the variant occurred de novo in the proband, while in eight cases (10%) it was inherited from the affected parent.

### 3.2. Association between Disease Severity, Variant Types and Nonsense-Mediated RNA Decay

**Figure 2:**
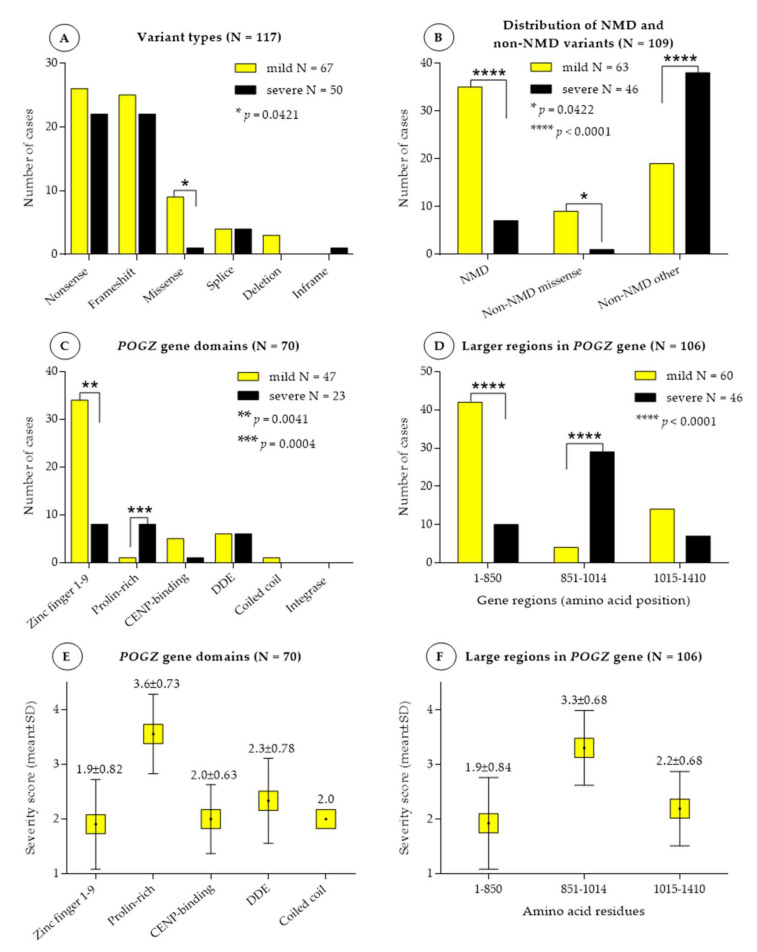
Distribution of all *POGZ*-variants in patients with mild and severe phenotypes. NMD: Nonsense-mediated RNA decay; CENP-binding: Centromere protein (CENP)-B-DNA-binding domain; DDE: originated from a transposase encoded by a pogo-like DNA transposon. Mild phenotype: patients with severity score 1 and 2, severe phenotype: severity score 3 and 4. (**A**): Distribution of the variant types in comparison to the severity of the phenotypes. Missense variants are significantly more frequent in mild phenotype than in severe (mild: 9, severe: 1, OR: 7.6, RR: 1.7). (**B**): Distribution of variant predicted to undergo NMD and those escaping NMD in comparison to the severity of the phenotypes. Non-NMD other indicates nonsense, frameshift variants and small in frame deletion. *NMD*: mild: 35, severe: 7, RR: 2.0, OR: 7.0; *non-NMD missense*: mild: 9, severe: 1, RR: 1.7, OR: 7.5. *Non-NMD other*: severe: 38, mild: 19, RR: 2.5, OR: 11. Splice variants excluded from analysis. (**C**): Distribution of mild and severe phenotypes in POGZ-domains. *Zinc finger 1–9:* mild: 34, severe: 8, RR: 1.7, OR: 4.9. *Prolin-rich*: severe: 8, mild: 1, RR: 3.6, OR: 25. (**D**): Distribution of mild and severe phenotypes in the larger *POGZ*-gene regions. Variants between *1–850 residues:* mild: 42, severe: 10, RR: 2.4, OR: 8.4, those between *851–1014 residues:* severe: 29, mild: 4, RR: 3.8, OR: 24. Splice variants and deletions excluded from analysis. (**E**): Mean ± SD of the severity scores of the variants in POGZ-domains. (**F**): Mean ± SD of the severity scores of the variants in the larger *POGZ*-gene regions.

A severity score was assigned to each variant based on the clinical features observed (Supplementary Table S1). Variants were grouped according to the variant type, their location within the gene, and whether they were predicted to undergo nonsense-mediated RNA decay (NMD) or escape NMD. The frequency of mild and severe phenotypes was compared with respect to different variant types, NMD or non-NMD variants, the different regions of the *POGZ* gene and also to the different domains of the protein (Figure 2).

Based on the variant types, no significant difference was found between the cohort with mild (severity score 1 and 2) and severe phenotype (severity score 3 and 4), except for the missense variants, which were significantly more often associated with milder phenotype than with severe one (*p* < 0.0421) (Figure 2A).

According to the predictions with NMDEscPredictor, a total of 42 frameshift and nonsense variants undergo NMD, which are located before the amino acid residue 810, and ultimately result in haploinsufficiency. The effect of whole *POGZ* gene deletion and deletion of exon 4–19 is supposed to be equal with that of nonsense-mediated RNA-decay. In whole gene deletion there is no mRNA-synthesis from the deleted allele, and deletion of exon 4–19 also results in a considerably truncated transcript, and most probably undergoes NMD as well. Thus, these three cases were also considered as variants affected by NMD. Splice variants were not included in the analysis. Overall, 67 variants, including nonsense and frameshift variants after amino acid residue 810, in frame deletion and all missense variants, are expected to escape NMD. NMD-affected variants, as well as NMD-escaping missense variants, are significantly more often associated with milder phenotype (*p* < 0.0001 and *p* = 0.0422, respectively), while NMD-escaping truncating variants are more often with severe phenotype (*p* < 0.0001) (Figure 2B, Supplementary Table S1).

### 3.3. Association between Disease Severity and POGZ Domains and Larger Gene Regions

**Figure 3:**
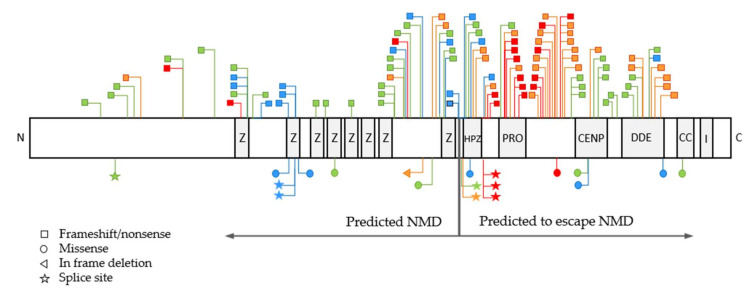
Distribution of variants in POGZ domains. NMD: nonsense-mediated RNA decay; CENP-binding: Centromere protein (CENP)-B-DNA-binding domain; DDE: originated from a transposase encoded by a pogo-like DNA transposon; Z: zinc finger domains 1-8; HPZ: HP1-binding zinc finger-like domain (zinc finger domain 9); CC: coiled coil domain; I: Integrase domain-binding motif. Severity score 1: blue, severity score 2: green, severity score 3: orange and severity score 4: red. Variant descriptions are detailed in Supplementary Table S1. Variants with discrepant labelling of severity (e.g., red-labeled variant in the N-terminal domain or zinc finger 1 domain) originate from cohort 3 with less detailed phenotypes.

Seventy variants occurred in the *POGZ* gene domains: ZF 1–9, CENPB, DDE and coiled coil domain and integrase domain-binding motif, while 36 variants occurred in between the domains. Deletions and splice variants were not included in the analysis. Between mild and severe phenotype, a significant difference was found in the zinc finger domain 1–9 and prolin-rich region (*p* = 0.041 and *p* = 0.0004, respectively). Variants in the prolin-rich region were associated with the most severe phenotypes (mean severity score: 3.6 ± 0.73). On the other hand, zinc finger domains were associated rather with mild phenotypes (mean severity score: 1.9 ± 0.82) (Figure 2C,E and Figure 3 and Supplementary Table S1). As several variants did not localize to the functional domains of POGZ, we then analyzed larger regions. Based on the distribution of severity scores, we categorized the variants in three larger regions: between amino acid residue 1–850 (encompassing ZF 1–9), 851–1014 (encompassing prolin-rich region) and 1015–1410 (encompassing CENPB, DDE, coiled coil and integrase domain). Similar to POGZ domains, variants in the region between 1–850 residues was associated with milder phenotype (mean severity score: 1.92) and variants in the region between 851–1014 residues with the most severe phenotypes (mean severity score: 3.30) (Figure 2D,F and Figure 3). One missense variant was detected in the region 850–1014 residues and was also associated with a severity score 4, suggesting that non-sense mediated decay and gene regions play a more crucial role in disease severity than variant types (Figure 3, Supplementary Table S1).

### 3.4. Frequency of POGZ-Related Symptoms

Facial dysmorphism was present in 96% of the patients analyzed from cohort 1 and 2. Among the facial features, the most common were hypertelorism (52%), midface hypoplasia (48%), broad forehead (44%), thin vermillion of the upper lip (42%), depressed flat nasal bridge (40%), tented mouth with downturned corners (40%) and epicanthus (38%) (Supplementary Table S1).

All patients had nervous system involvement. The most common symptoms were speech delay in 88%, global developmental delay in 88%, intellectual disability in 79%, seizures in 60% and sensorineural hearing impairment in 54% of the patients. Behavioral abnormalities were reported in 75%, and among those individuals 42% of the patients showed limited social interactions and 35% had autism spectrum and anxiety disorder (Supplementary Table S1).

Ocular anomalies were diagnosed in 63% of the patients among which strabismus was the most common symptom in 25%, followed by astigmatism and optic nerve hypoplasia with 15%.

Muscular hypotonia and sleep disturbances were also frequently observed, in 54% and 75% of the patient cohort, respectively. Microcephaly was noted in 46%, brachycephaly in 35%, both short neck and short stature in 29%, brachydactyly and small hands in 23% and joint laxity also in 23% (Supplementary Table S1).

A tendency to obesity could be observed in 42% (BMI ≥ 97th percentile), while early feeding difficulties in 23% of the patients. Perinatal complications were reported in 17%, genitourinary tract anomalies in overall 31% of the patients, where one of the leading anomalies was duplicated renal collecting system in 13% (Supplementary Table S1).

A clear tendency in the evolution of the clinical picture could not yet been identified, since only three patients older than 30 years were reported until present. One patient (G01) received the diagnosis of autism spectrum disorder in adulthood, although worsening of her condition has not been mentioned, while another patient (ID 92) presented the worsening of strabismus with age and the development of obsessive-compulsive disorder and bipolar disorder diagnosed in adulthood, following the initial diagnosis of autism spectrum disorder (Supplementary Table S1) [29].

### 3.5. Facial Gestalt Analysis

**Figure 4:**
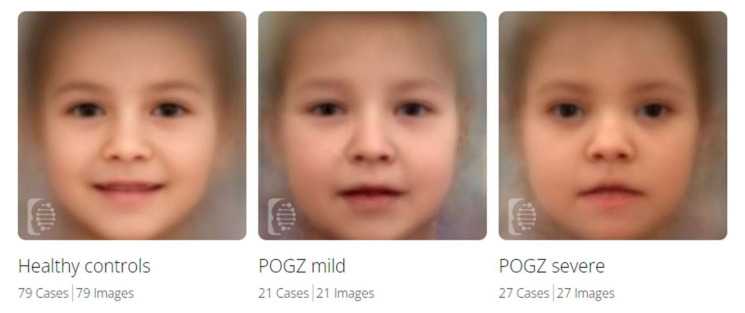
Composite images from Face2Gene for *POGZ* patients with mild and severe phenotype and healthy controls.

We used the Face2Gene Software to contrast cohorts comprised of patients with different disease severity and controls. Composite images of *POGZ* patients with mild (severity score 1 and 2) and severe phenotype (severity score 3 and 4) and healthy controls showed the facial differences between the three cohorts (Figure 4). *POGZ* patients have in general hypertelorism, broad forehead, broad nasal bridge, downturned corners of the mouth, cupid′s bow of the upper lip and midface hypoplasia. These features are more prominent in patients with severe phenotype than mild.

The software found marked differences between healthy individuals and *POGZ* patients with mild phenotypes (*p* < 0.001), and also between healthy individuals and *POGZ* patients with severe phenotypes (*p* < 0.001). A difference could be observed between individuals with mild and severe phenotypes, but did not rise to statistical significance (Table 4).

## 4. Discussion

White-Sutton syndrome represents a wide and variable spectrum of symptoms and abnormalities related to variants or copy number variations in *POGZ* gene. A clear association between variants and clinical severity has not yet been established [32].

To our knowledge, our study is the largest systemic genotype-phenotype comparison in White-Sutton syndrome, which uses an integrated severity score for each patient based on a detailed clinical scoring system. The same variants from different studies received similar severity score, which indicates that the clinical scoring system was well-established and correctly unified despite the differently elaborated clinical details of the published cases. However, biased severity scores may still be present as a result of missing clinical information from cases with less detailed phenotypes, or differences in the clinical assessments from different studies.

Overall, 117 patients with 72 different variants were enrolled in the analysis, including eight novel variants. The majority of the variants were nonsense (41%) or frameshift (40%), but missense (8.5%), splice site variants (7%), being small in frame and larger deletions (3.5%) were also observed.

Previous studies suggested that missense variants are associated with milder phenotypes, such as autism spectrum disorder and other neuropsychiatric conditions [14,18,22,25,33], while nonsense and frameshift variants are associated with more severe phenotypes [5]. In our cohort, missense variants were also more often associated with milder phenotypes, however one missense variant in the prolin-rich region presented with a higher severity score. This suggests that variant type may have a lesser effect on the clinical outcome than the presence or absence of nonsense-mediated RNA decay or the location of the variants when having escaped NMD (Figure 2 and Figure 3).

Patients whose variants were predicted to undergo NMD presented with milder phenotypes. A potential explanation may be that NMD of the aberrant mRNAs resulted in haploinsufficiency of the POGZ protein, but the rest of the normal mRNAs were translated and could compensate for this loss, thus resulting in a milder phenotype with less dysmorphic features and more mild neurocognitive impairment, such as developmental delay, borderline-mild intellectual disability and behavioral issues, than structural malformations of the brain or other organ systems. This is consistent with the results of Stessman and colleagues, showing that in POGZ ortholog knockdown Drosophila flies, the plastic behavioral response was affected without severe neurological defects [6].

In contrast, variants escaping NMD could result in the translation of the aberrant mRNAs and lead to deleterious gain-of-function or dominant-negative activity of the resulting truncated protein. Such aberrant gain of function or dominant negative activity may impair the activity of the normal allele, and culminate in a more severe phenotype. This is also supported by the fact that most of the potentially pathogenic variants fall within the second half of the protein (Figure 3) [5,6,25,30].

To date, the most important function attributed to POGZ protein is its binding to heterochromatin protein 1α. HP1α may bind to several heterochromatin protein binding proteins (HPBPs): canonical HPBPs with PxVxL motives, such as Aurora B and INCENP—components of chromosome passenger complex (CPC), kinetochore proteins and cohesion-related proteins, as well as proteins of the histone methyltransferase complex and zinc finger proteins, including POGZ. In interphase cells HP1α is located on the chromosome arm, attached to histone 3 (H3K9) and bound to CPC, which inhibits gene expression. During progression into mitosis, a specific zinc finger domain of the POGZ protein (HPZ) binds to HP1α, competing with the CPC proteins for the binding site on HP1α, and this leads to the detachment of HP1α from the chromosome arm. Beside the dissociation of HP1α from the chromosome arm, normal POGZ protein also promotes the correct localization of CPC in the centromeric region, and increases the phosphorylation of histone 3 protein by triggering the kinase activity of CPC. Mutations disabling the HPZ function of POGZ protein abolished its interaction with HP1α. In human POGZ knockdown cells (reduced POGZ protein level in HeLa cells treated with POGZ siRNA) HP1α and CPC remained on the chromosome arms during mitosis, an impaired mitotic progression with mitotic delay, chromosome misalignment and abnormal chromosome segregation could be observed [1]. This also suggests that the effect of mutations undergoing NMD and resulting in haploinsufficiency may differ from that of NMD-escaping mutations with intact HPZ domain.

This, on the other hand, drew attention to the localization of the variants and to the importance of POGZ domains not affected by NMD. To date, only the function of HPZ (NMD-affected), CENBP and DDE domains (NMD-escaping) has been investigated. HPZ was proved to be essential for the chromatin binding and its variants abolished the interactions with HP1α [1], thus impairing the chromatin accessibility and gene expressions [3]. Variants in CENBP domain impaired the nuclear localization of POGZ protein, disrupted its DNA-binding activity, impaired cortical differentiation and increased the activity of excitatory cortical neurons in mice [2,34]. DDE was suggested to interact with transcriptional coactivators (LEDGF/p75) involved in the neuroepithelial stem cell differentiation and neurogenesis [6,35,36].

Our analysis showed that nonsense, frameshift (destroyed by NMD) and missense variants located in the first half of the protein (including ZF 1–8 and HPZ) are associated with milder phenotypes, while variants (NMD-escaping nonsense, frameshift and also missense) in the prolin-rich domain and in its close proximity cause the most severe outcomes. The distal part of the protein, including CENPB, DDE and coiled coil domain, were associated with a mild-to-moderate disease severity. In the C-terminal end of POGZ (integrase domain-binding motif), no pathogenic variants have been identified yet (Figure 3).

Clear association between variants in the HPZ domain and clinical phenotype could not be made. The HPZ domain contained only seven variants (one missense and six truncating), all of which were predicted to survive NMD. Only two variants, located closer to the prolin-rich region, were associated with a higher severity score (Figure 3, Supplementary Table S1). Thus, we suppose that variants located in the prolin-rich domain or in its proximity may have a more deleterious impact on the protein function than variant in the HPZ or CENBP domain. HPZ and CENBP variants may result in the loss of chromatin binding function, due to either NMD or impaired nuclear localization, while prolin-rich region may potentially trigger other mechanisms, such as a dominant negative effect or gain of function. However, a final conclusion could not be made due to the relatively low number of reported variants in these regions, and the lack of further variant-specific functional studies.

The neurocognitive abnormalities with different severity were present in all analyzed *POGZ* patients. Facial dysmorphic features were also detectable in the great majority of patients, although the extent of facial dysmorphism was different between patients with milder and severe phenotype (Figure 4). Behavioral abnormalities, skeletal anomalies, disorders of the gastrointestinal tract and ocular system also belonged to the major symptoms of White-Sutton syndrome. This was in good concordance with a previous review [32].

In conclusion, we suggest the use of the detailed clinical scoring system developed in this study for the evaluation of *POGZ* patients. However, fine-tuning of the scoring system, such as prioritizing the clinical features or ruling out some minor ones may be necessary for a more accurate prediction of severity. Functional studies on nonsense-mediated RNA decay and domain functions are nevertheless crucial to unravel the molecular pathophysiology underlying the diversity of *POGZ*-related disorders.

## Figures and Tables

**Table 1:** Clinical scoring system for the phenotypic features in *POGZ*-patients.

1. DYSMORPHIC FACIAL FEATURES	CLINICAL SCORES
Broad/high forehead/bitemporal narrowing	Scoring from 1 to 4 (mild-to-severe facial dysmorphism) <5 features: 1 scoring point 5–10 features: 2 scoring point 11–15 features: 3 scoring point >15 features: 4 scoring point
Hypertelorism	
Downslanting or upslanting palpebral fissures	
Epicanthus	
Ptosis	
High-arched/sparse eyebrows	
Broad nasal tip	
Depressed, flat nasal bridge	
Pear-shaped nose	
Midface hypoplasia/retrusion	
Short philtrum	
Downturned corners of mouth (triangular/tented)	
Upper lip (cupid’s bow)	
Thin vermillion/thin upper lip	
Everted upper/lower lip	
Open mouth	
Macrostomia	
Protrusion of the tongue/macroglossia	
High-arched palate	
Bifid uvula	
Mandibula (prognathia or micro-retrognathia)	
Pointed chin	
Low-set ears	
Posteriorly rotated ears	
Over-folded/abnormally folded helices	
**2. ABNORMALITY OF THE EYE**	
Strabismus	1 scoring point/symptom
Myopia	
Hypermetropia	
Anisometropia	
Astigmatism	
Iris coloboma	
Optic nerve atrophy or hypoplasia	
Rod-cone dystrophy	
Cortical visual impairment	
Abnormal electroretinogram	
Abnormal visual evoked potentials	
**3. ABNORMALITY OF THE NERVOUS SYSTEM**	
Global developmental delay	Developmental delay and intellectual disability were scored from score 1 (+, mild) to score 4 (++++, severe) based on the *MEAN* of the scoring points given for gross motor (A), speech delay (B) and IQ-level (C) (Details seen in Supplementary Table S1): ***A.*** Age at walking 16–23 months: + 24–30 months: ++ 31–48 months: +++ >48 months: ++++ ***B.*** Age at talking (talking in simple sentences) 18–23 months: + 24–36 months: ++ 37 months–5 years: +++ >5 years/no speech: ++++ ***C.*** Intellectual disability Borderline: + Mild: ++ Moderate: +++ Severe: ++++
Gross motor developmental delay	
Age at walking	
Fine motor developmental delay	
Speech delay/No speech	
Age at talking	
Receptive language disorder	
Expressive language disorder	
Intellectual disability (IQ, if applicable)	
Aplasia/hypoplasia of the corpus callosum	Additional scores: 1 scoring point/CNS abnormalities and additional neurological symptoms Only 1 scoring point in the presence of both seizures and EEG-abnormalities No score for antiepileptics
Cerebral atrophy	
Polymicrogyria/simplified gyral pattern	
Brainstem hypoplasia	
Cerebellar dysplasia/hypoplasia	
Periventricular white matter lesion	
Delayed myelination	
Optic chiasma dysplasia	
Dandy-Walker malformation/variant	
Ventriculomegaly	
Other central nervous system (CNS) abnormality	
Sensorineural hearing loss (bilateral/unilateral)	
Seizures	
EEG abnormality	
Hypoglycemic seizures	
Febrile seizures	
Antiepileptics (mono therapy/combined)	
**4. BEHAVIORAL ABNORMALITIES**	
Autism spectrum disorder	1 scoring point/behavioral abnormality
(Self-)injurious behavior	
Anxiety	
Attention deficit hyperactivity disorder	
Limited social interactions	
Low frustration tolerance (tantrums)	
**5. ABNORMALITY OF THE MUSCULATURE**	
Hypotonia (facial, axial, appendicular, generalized, others)	0 scoring point: no hypotonia/not reported 1 scoring point: if any type of hypotonia was reported
**6. NORMALITY OF THE CARDIOVASCULAR SYSTEM**	
Congenital heart defect	1 scoring point/cardiovascular defect
Atrial septal defect	
Persistent ductus arteriosus	
**7. ABNORMALITY OF THE SKELETAL SYSTEM**	
Brachycephaly	1 scoring point/skeletal abnormality
Microcephaly	
Plagiocephaly	
Head circumference in cm (percentile/-SD)	
Cleft palate	
Short neck	
Brachydactyly/Small hands	
Syndactyly	
Broad fingers and toes	
Clinodactyly	
Joint laxity	
Scoliosis	
Contractures	
Short stature	
Skeletal anomalies of the lower extremities	
**8. ABNORMALITY OF THE DIGESTIVE SYSTEM**	
Feeding difficulties: dysphagia, swallowing difficulty	1 scoring point/gastrointestinal abnormality
Tube feeding/Gastrostomy tube	
Gastroesophageal reflux	
Constipation	
Cyclic vomiting	
Failure to thrive	
Overweight/Obesity	
Diaphragmatic hernia	
Other hernias	
Intestinal malrotation, intussusception	
Rectal prolapse	
**9. PERINATAL MEDICAL HISTORY**	
Prenatal or postnatal complications and findings (high nuchal translucency, low Apgar scores, microcephaly, etc.)	0 scoring point: no prenatal/perinatal problem or not reported 1 scoring point: if any type of problem was reported
**10. GENITO-URINARY TRACT ABNORMALITY**	
Duplicated renal collecting system	1 scoring point/genito-urinary abnormality
Ureteropelvic junction obstruction	
Renal dysplasia	
Cryptorchidism	
Hypoplastic scrotum	
Hypoplastic testes	
Micropenis	
Phimosis	
Primary amenorrhea	
**11. MISCELLANEOUS**	
Sleep disturbance (obstructive sleep apnea)	1 scoring point/abnormality
Frequent respiratory infections	
Recurrent otitis media	
Others	
**CUMULATIVE CLINICAL SCORE:**	*SUM* of the scores given to organ system/category 1–11

The detailed clinical scoring for each patient is presented in Supplementary Table S1. In the section of nervous system abnormalities + indicates the severity in the Supplementary Table S1: +: 1 scoring point, ++: 2 scoring points, +++: 3 scoring points, ++++: 4 scoring points. In patients with less clinical details, dysmorphic features were not evaluated for the clinical score due to the lack of clinical information or photos of patients.

**Table 2:** Established disease severity scores based on the cumulative clinical scores in the three different patient cohorts.

SEVERITY SCORES	CUMULATIVE CLINICAL SCORES IN:		
	Our Patients	Published Cases with Detailed Phenotypes	Published Cases with Less Detailed Phenotypes
**1**	1–10	<9	1–3
**2**	11–20	9–14	4–6
**3**	21–30	15–19	7–10
**4**	≥31	≥20	≥11

Severity score 1: the mildest manifestation of the disease; score 2: moderate; score 3: moderate-severe; score 4: the most severe manifestation of the disease.

**Table 3:** Genotypes, cumulative clinical scores and disease severity scores in our *POGZ* cohort (N = 13).

Patient ID	Age at Last Follow-Up/Age at the Diagnosis /Gender	Variant in *POGZ* Gene	ACMG Classification ** and ClinVar Submissions/ Frequency in Gnomad	*De Novo*	Ethnicity	Cumulative Clinical Scores	Severity Score
**L01**	2 ys/11 months/male	**c.2873_2874delCA;** p.Ala958Valfs*6	Path (PVS1, PM2, PM6)/0	de novo	Caucasian	53	**4**
**L02**	6.5 ys/4 ys /female	**c.2763del;** p.Thr922Leufs*6	Path (PVS1, PM2, PM6, PP3)/0	de novo	Caucasian	31	**4**
**G01**	35 ys/35 ys /female	c.1522C>T; p.Arg508 *	Path (PVS1, PM2, PP3) ClinVar +/0	unknown	Caucasian	9	**1**
**G02**	5 ys/5 ys /male	c.1522C>T; p.Arg508 *	Path (PVS1, PM2, PP3) ClinVar +/0	maternal	Caucasian	7	**1**
**W01**	11 ys/9 ys /female	**c.2190T>G;** p.Tyr730 *	Path (PVS1, PM2, PM6, PP3)/0	de novo	Caucasian	16	**2**
**S01**	5 ys/4.2 ys	c.3259C>T; p.Arg1087*	Path (PVS1, PM2, PM6, PP3, PP5) ClinVar + + /0	de novo	Caucasian	13	**2**
**D01**	17 ys/17 ys /male	**c.2258G>A;** **p.Cys753Tyr**	VUS (PM2, PP3, PM6)/0	de novo	Caucasian	11	**2**
**R01**	8 ys/7 ys /male	**c.600dupT;** p.Gly201Trpfs*114	Path (PVS1, PM2, PM6, PP3)/0	de novo	Caucasian	22	**3**
**R02**	3 ys/2.5 ys /male	**c.2103delT;** p.Pro701fs*64	Path (PVS1, PM2, PM6)/0	de novo	Caucasian	18	**2**
**US01**	14 ys/14 ys /male	c.1180_1181delAT; p.Met394Valfs*9	Path (PVS1, PM2, PM6, PP3, PP5) ClinVar +/0	de novo	Caucasian	13	**2**
US02 *	1 month/1 month/female	c.2545G>T; p.Gly849 *	Path (PVS1, PM2, PM6, PP3)/0	de novo	Caucasian	3	**1**
**US03**	2 ys 5 months/ 2 months/male	**c.3196A>T;** p.Lys1066 *	Path (PVS1, PM2, PP3)/0	unknown	Caucasian	12	**2**
**NL01**	7 ys/7 ys	**Deletion 1q21.3** **encompassing the whole *POGZ* gene**		de novo	Caucasian	16	**2**

Novel variants are written in bold. * Patient has been previously involved in a study about congenital diaphragm hernia. ** Effect of the variant was predicted *in silico* by using Varsome, based on the classification of ACMG Guidelines: PVS1: very strong evidence of pathogenicity, PM1-6: moderate evidence, PP1-5: supporting evidence [9]. Path: pathological, VUS: variant of unknown significance, prediction based on ACMG criteria. In patient US02 the severity score may be incorrect due to the young age of the patient and lack of clinical details. The severity score was assigned based on current features but may evolve over time. Scale of disease severity scores: 1 (the mildest) to 4 (the most severe). *POGZ* transcript: NM_015100.

**Table 4:** Binary comparison of the facial features of *POGZ*-cohorts with mild and severe phenotypes by using Face2Gene software.

BINARY COMPARISON	NO OF CASES	MEAN AUC	AUC SD	*P* VALUE FOR AUC
**Healthy vs. Mild**	79 vs. 21	0.90	0.04	<0.001
**Healthy vs. Severe**	79 vs. 27	0.96	0.02	<0.001
**Mild vs. Severe**	21 vs. 27	0.74	0.06	0.067

AUC: area under the curve, SD: standard deviation. A *p* value <0.05 represents a high degree of discrimination.

## Data Availability

All data are available in the article or in the Supplementary Materials.

## Supplementary Materials

The following supporting information can be downloaded at https://www.mdpi.com/article/10.3390/genes13010154/s1. Supplementary Table S1: Part A: Clinical features of 13 *POGZ* patients. Part B: Clinical features of *POGZ* patients with detailed clinical information. Part C: Clinical features of *POGZ* patients with less detailed clinical information. Part D: Detailed genotype-phenotype comparison. Part E: Summarized data for statistics. Part F: Frequency of clinical symptoms.
